# Assembly of lipase and P450 fatty acid decarboxylase to constitute a novel biosynthetic pathway for production of 1-alkenes from renewable triacylglycerols and oils

**DOI:** 10.1186/s13068-015-0219-x

**Published:** 2015-02-26

**Authors:** Jinyong Yan, Yi Liu, Cong Wang, Bingnan Han, Shengying Li

**Affiliations:** Key Laboratory of Biofuels, and Shandong Provincial Key Laboratory of Energy Genetics, Qingdao Institute of Bioenergy and Bioprocess Technology, Chinese Academy of Sciences, No. 189 Songling Road, 266101 Qingdao, Shandong China; University of Chinese Academy of Sciences, No. 19A Yuquan Road, 100049 Beijing, China; Key Laboratory for Marine Drugs, Department of Pharmacy, Renji Hospital, Shanghai Jiao Tong University School of Medicine, No. 145 Shandongzhong Road, 200127 Shanghai, China

**Keywords:** Lipase, P450 fatty acid decarboxylase, Alkenes, Cell-free catalysis, Whole cell catalysis

## Abstract

**Background:**

Biogenic hydrocarbons (biohydrocarbons) are broadly accepted to be the ideal ‘drop-in’ biofuel alternative to petroleum-based fuels due to their highly similar chemical composition and physical characteristics. The biological production of aliphatic hydrocarbons is largely dependent on engineering of the complicated enzymatic network surrounding fatty acid biosynthesis.

**Result:**

In this work, we developed a novel system for bioproduction of terminal fatty alkenes (1-alkenes) from renewable and low-cost triacylglycerols (TAGs) based on the lipase hydrolysis coupled to the P450 catalyzed decarboxylation. This artificial biosynthetic pathway was constituted using both cell-free systems including purified enzymes or cell-free extracts, and cell-based systems including mixed resting cells or growing cells. The issues of high cost of fatty acid feedstock and complicated biosynthesis network were addressed by replacement of the *de novo* biosynthesized fatty acids with the fed cheap TAGs. This recombinant tandem enzymatic pathway consisting of the *Thermomyces lanuginosus* lipase (Tll) and the P450 fatty acid decarboxylase OleT_JE_ resulted in the production of 1-alkenes from purified TAGs or natural oils with 6.7 to 46.0% yields.

**Conclusion:**

Since this novel hydrocarbon-producing pathway only requires two catalytically efficient enzymatic steps, it may hold great potential for industrial application by fulfilling the large-scale and cost-effective conversion of renewable TAGs into biohydrocarbons. This work highlights the power of designing and implementing an artificial pathway for production of advanced biofuels.

**Electronic supplementary material:**

The online version of this article (doi:10.1186/s13068-015-0219-x) contains supplementary material, which is available to authorized users.

## Introduction

Shortage of petroleum-based fuels and increasing environmental concerns have led to great efforts to develop sustainable and clean biofuels from renewable feedstocks. Bioethanol manufactured by fermentation of sugars and biodiesel produced *via* transesterification of vegetable oils, animal fat, or waste oils are regarded as the two major first-generation biofuels, which are dominating the current global market of biofuels [1]. However, some undesirable properties such as high miscibility with water and low energy density (for bioethanol), incompatibility with current engine systems, and problems associated with storage and distribution have limited their further popularization [2]. Therefore, advanced biofuels with better fuel properties have been receiving ever-increasing attention, among which biohydrocarbons, especially the medium- to long-chain fatty alkanes/alkenes, are considered the ideal alternatives to petroleum-based transportation fuels due to their highly similar chemical composition and physical characteristics. Thus, biohydrocarbons hold the potential to be developed into ‘drop-in’ biofuels compatible with the existing distribution infrastructures used for gasoline, diesel, and jet fuels in terms of their fatty acyl chain length [3].

At present, chemical hydrotreatment of acylglycerides, fatty acids, or fatty acyl esters, and *de novo* microbial biosynthesis are the two major strategies for production of biohydrocarbons. The former strategy requires expensive metal catalysts (for example, Pd and Pt), high temperature (250 to 450°C), and high pressure (20 to 70 bar), hence being energy intensive and environmentally unfriendly [4]. In comparison, the latter bioproduction strategy highlighted by a limited number of metabolically engineered systems is undoubtedly ‘greener’ [5-10]. However, all these biosynthetic systems suffer greatly from low yield of hydrocarbons, preventing them from industrialization.

For instance, the first engineered alkane biosynthetic pathway consisting of the acyl-acyl carrier protein (ACP) reductase Orf1594 from *Synechococcus elongates* PCC7942 and the aldehyde decarbonylase from *Nostoc punctiforme* PCC73102 was constructed in *Escherichia coli* and gave rise to a total alkane titer of approximately 300 mg/l [5]. Later, two similar hybrid biosynthetic routes by coupling a carboxylic acid reductase or a fatty acid reductase complex to the same fatty aldehyde decarbonylase were also reconstituted, however, leading to lower alkane yields [6,7]. Recently, based on the *in vivo* activity of the long-chain fatty acid P450 decarboxylase OleT_JE_, our laboratory engineered a series of *E. coli* strains capable of *de novo* biosynthesis of 1-alkenes from glucose with the highest total alkene titer of 96.7 mg/l [10].

**Figure 1:**
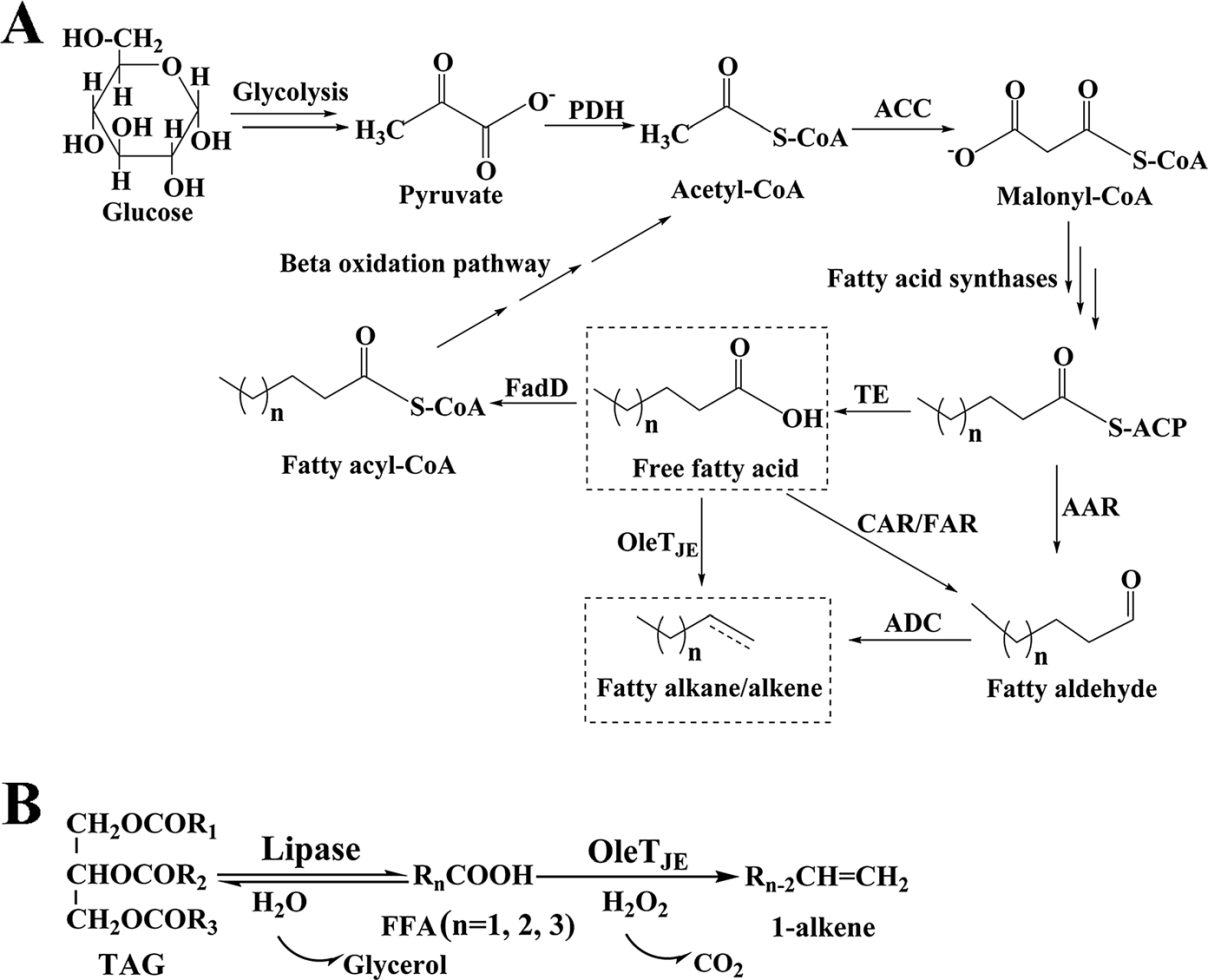
**Multi-step and artificial two-step pathways. (A)** Native and engineered multi-step pathways for hydrocarbon biosynthesis based on fatty acid metabolic network. **(B)** The artificial two-step 1-alkene biosynthetic pathway developed in this work consisting of the lipase Tll and the P450 fatty acid decarboxylase OleT_JE_. AAR, acyl-ACP reductase; ACC, acetyl-CoA carboxylase; ACP, acyl carrier protein; ADC, aldehyde decarbonylase; CAR, carboxylic acid reductase; FAR, fatty acid reductase; PDH, pyruvate dehydrogenase; TAG, triacylglycerol; TE, thioesterase.

All these engineered pathways were constructed through diverting the fatty acid biosynthetic pathway toward hydrocarbon synthesis via different mechanisms of deoxygenation (Figure 1A). Since fatty acid biosynthesis plays a central role in energy metabolism of all living organisms and involves multiple interplaying enzymatic steps [11-17], its regulation is highly complicated and hence difficult to be manipulated. Metabolic engineering efforts aimed at overproduction of fatty acids or their derivatives (for example, fatty acyl-ACPs or fatty acyl CoAs) for high production of hydrocarbons *via* genetically reprogramming the fatty acid biosynthetic system turn out to be intrinsically difficult and technically challenging.

To overcome the limit of *de novo* biosynthesis of hydrocarbons, in this study, we propose a novel strategy for long-chain fatty alkene biosynthesis through coupling the glyceride hydrolytic activity of the *Thermomyces lanuginosus* lipase (Tll) to the fatty acid decarboxylation activity of the P450 enzyme OleT_JE_ (Figure 1B). Tll has been well characterized as a biocatalyst with remarkable capacity of hydrolyzing triacylglycerols (TAGs) to free fatty acids (FFAs) involved in oil modifications [18]. OleT_JE_ was recently discovered as a novel P450 enzyme with the unique ability to decarboxylate long-chain FFAs, forming terminal alkenes (1-alkenes) [19]. It is evident that FFAs should be able to couple these two naturally unrelated reactions. Thus, we selected these two enzymes to construct an artificial two-step biosynthetic pathway for biological production of 1-alkenes.

In the pilot biosynthetic system based on purified enzymes, the exogenous feedstock including pure TAGs and natural oils were first efficiently hydrolyzed by Tll, and the released free fatty acids (FFAs) were decarboxylated by OleT_JE_ resulting in 1-alkenes (Graphical abstract in Additional file 1). Upon the proof-of-concept by using pure enzymes, the cell-based systems including the mixed resting cells that express Tll and OleT_JE_, respectively, and the resting cells that co-express Tll and OleT_JE_ were also evaluated. Moreover, the *in situ* catalytic system that is capable of mediating the biotransformation of TAGs → FFAs → 1-alkenes during cell growth was further customized from the perspective of cost-effective production of alkenes at industrial scale. This innovative tandem biotransformation process for producing olefin hydrocarbons from TAGs broadens the application of lipase in biofuel synthesis, which has long been only focused on biodiesel production [20-22]. The simple two-step (hydrolysis followed by decarboxylation) enzymatic pathway appears to be efficient and more manageable compared to the complex fatty acid biosynthetic network. Since TAGs are widely present in plant oils, animal fats, and oleaginous microbial cells [23], the feedstock for future large-scale application of this new biohydrocarbon-producing strategy should be abundant and cost-effective.

## Results and discussion

### Conversion of TAGs → FFAs → 1-alkenes catalyzed by purified Tll and OleT_JE_

**Figure 2:**
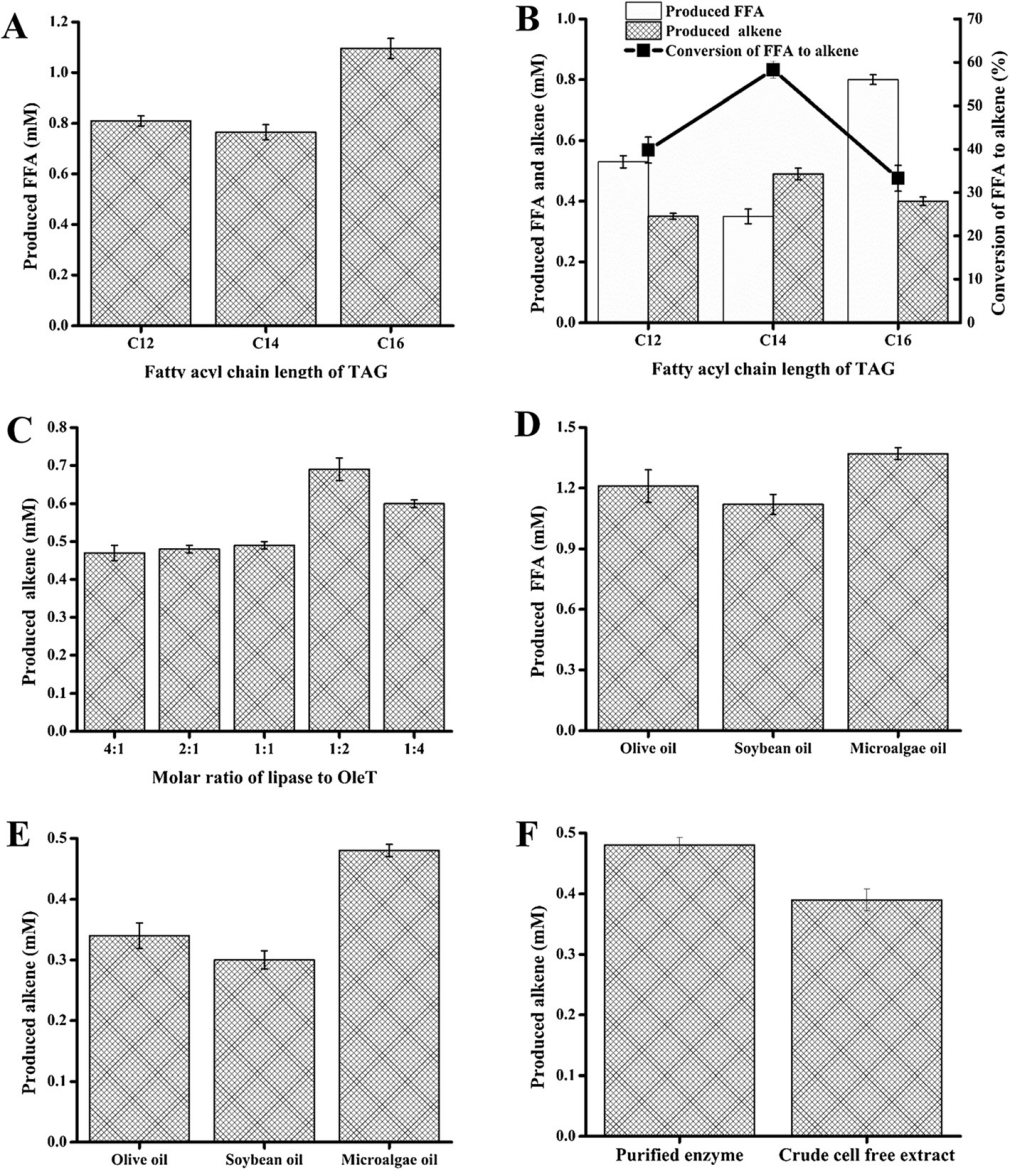
**Production of FFAs and 1-alkenes and optimization of the enzyme ration between Tll and OleT**_**JE**_ **. (A)** Production of FFAs from hydrolysis of different TAGs by purified Tll. **(B)** Production of FFAs and 1-alkenes from TAGs catalyzed by the coupled Tll-OleT_JE_. **(C)** Optimization of the enzyme ratio between Tll and OleT_JE_ for improvement of the overall alkene yield from the TAG with C14 fatty acyl chain length. **(D)** Production of FFAs from hydrolysis of different natural oils by purified Tll. **(E)** Production of 1-alkenes from natural oils catalyzed by the coupled Tll-OleT_JE_. **(F)** Production of 1-alkenes from microalgae oil mediated by purified enzymes and cell-free extracts. FFA, free fatty acid; TAG, triacylglycerol.

The two enzymes OleT_JE_ and Tll were functionally expressed in *E. coli* and purified to homogeneity (Figure S1 in Additional file 2). In the lipase-catalyzed hydrolysis of pure TAGs (0.5 mM) that only have C12, C14, and C16 fatty acyl chains (that is, trilaurin, trimyristin, and tripalmitin), Tll released 0.80 mM lauric acid (C12), 0.78 mM myristic acid (C14), and 1.15 mM palmitic acid (C16), corresponding to 53.3 ± 1.3, 52.0 ± 1.1, and 76.7 ± 1.5% yield (Figure 2A), respectively.

To test whether the fatty acid decarboxylation activity of OleT_JE_ can be coupled to the lipase-catalyzed hydrolysis in one pot, 2 μM OleT_JE_ and 1 mM H_2_O_2_ as cofactor were added into the TAG hydrolytic reactions. As expected, 0.35 mM 1-undecene (C11), 0.49 mM 1-tridecene (C13), and 0.40 mM 1-pentadecene (C15) were produced (Figure 2B), corresponding to 23.3 ± 0.9, 32.7 ± 1.4, and 26.7 ± 0.9% of the theoretically maximal conversion from TAG to corresponding 1-alkene. When only taking the second decarboxylation step into account, as shown in Figure 2B, the conversions of the C12, C14, and C16 FFA to corresponding 1-alkene were 39.8 ± 1.5, 58.3 ± 1.3, and 33.3 ± 1.1%, respectively. These results evidently demonstrate the compatibility between the reactions mediated by Tll and OleT_JE_. Among the tested TAG hydrolysis reactions, Tll displayed the highest activity toward tripalmitin (C16 TAG), which indicates the fatty acyl chain length preference of Tll being C16 over C14 and C12. By contrast, in the tandem hydrolysis-decarboxylation process, the alkene product with the greatest yield turned out to be 1-tridecene (Figure 2B), decarboxylated from the C14 FFA-myristic acid. In the one-pot catalytic relay, the consumption of fatty acids by OleT_JE_ (forming alkenes) was expected to pull the equilibrium of TAGs ↔ fatty acids toward fatty acids (Figure 1B). Consistently, the total FFA yield of the tested dual enzymatic systems calculated by summing up the reacted FFA (equals to the produced alkene in mole) and the remaining FFA (Figure 2B) was slightly improved compared to that of the single lipase hydrolytic system (Figure 2A).

In a mixed biocatalytic system of dual enzymes, the ratio between the two enzymes is often a key factor for the overall conversion ratio. Thus, we elected to use trimyristin (the TAG with three C14 fatty acyl chains), which gave the highest 1-alkene yield at a 1:1 enzyme ratio (Figure 2B), as substrate to optimize the proportion of lipase to decarboxylase. As shown in Figure 2C, the C13 alkene yield was improved to 0.69 mM (corresponding to 46.0 ± 1.8% conversion of TAG to alkene) at a molar ratio of 1:2 (Tll:OleT_JE_). It was reported that the extensive metabolic engineering efforts aimed at overproduction of FFAs in *E. coli* have so far achieved less than 30% of the maximum theoretical yield *via de novo* biosynthesis from the starting carbon source glucose [16]. The reported yields of FFA downstream products such as hydrocarbons were even lower [5-7,10]. Thus, the 46.0% overall conversion from TAG to 1-alkene achieved by the cell-free system of tandem lipase-OleT_JE_ in this work appears significant. The *in vitro* manipulation *via* enzyme ratio optimization held another significant advantage over the less accurate *in vivo* metabolic engineering, which likely requires a delicate coordination of the complicated regulation networks of transcription, translation, and metabolite fluxes.

Subsequently, three natural oils other than pure TAGs including olive oil, soybean oil, and microalgae oil were investigated as substrates for the novel Tll-OleT_JE_ catalytic relay system. The Tll-catalyzed hydrolysis gave rise to 1.21, 1.12, and 1.37 mM total FFAs from olive oil, soybean oil, and microalgae oil (Figure 2D), corresponding to 80.7 ± 2.3, 74.7 ± 2.1, and 91.3% ± 3.3 conversion of TAGs to FFAs, respectively. The profiles of produced FFAs are described in Table S1 in Additional file 3. In the coupled hydrolysis-decarboxylation reactions, 0.34, 0.30, and 0.48 mM total alkenes including 1-tridecene (for microalgae oil only), 1-pentadecene, and 1-heptadecene, corresponding to 21.3 ± 0.7, 20.0 ± 0.9, and 32.0 ± 1.0 overall conversion from TAGs to alkenes (Figure 2E; Table S1 in Additional file 3), were produced from olive oil, soybean oil, and microalgae oil, respectively. Interestingly, no higher alkenes were observed in this coupled reaction system, although FFAs of C20:5 and C22:6 were released from microalgae oil. This indicates the relatively lower substrate flexibility of OleT_JE_ than that of Tll, which is consistent with the reported fatty acid chain length selectivity of saturated C12 to C20 [10,19]. Comparatively, microalgae oil appeared to be a more suitable feedstock for 1-alkene production *via* tandem biocatalysis. Since microalgae oils could be an excellent feedstock for this biosynthetic pathway to produce advanced hydrocarbon biofuels, all following experiments used this feedstock.

To reduce the biocatalyst cost by omitting the enzyme purification step, the crude *E. coli* cell-free extracts of Tll and OleT_JE_ were applied for the production of 1-alkene using microalgae oil as feedstock. As a result, 0.39 mM 1-alkenes were generated, comparable to the 0.48 mM yield when using purified enzymes (Figure 2F).

### Production of 1-alkenes mediated by mixed resting *E. coli* recombinant cells

Biotransformation mediated by whole cell catalysts represents a promising strategy for industrial manufacture because it skips the costly enzyme purification and immobilization steps. To reduce the cost on biocatalysts, the two *E. coli* whole cell catalysts of Tll and OleT_JE_ were mixed and evaluated. Importantly, in the mixed cell system, the enzyme ratio can be easily controlled by adjusting cell dosage. However, multiple events of substrate/product transfer across cell membrane must be considered.

**Figure 3:**
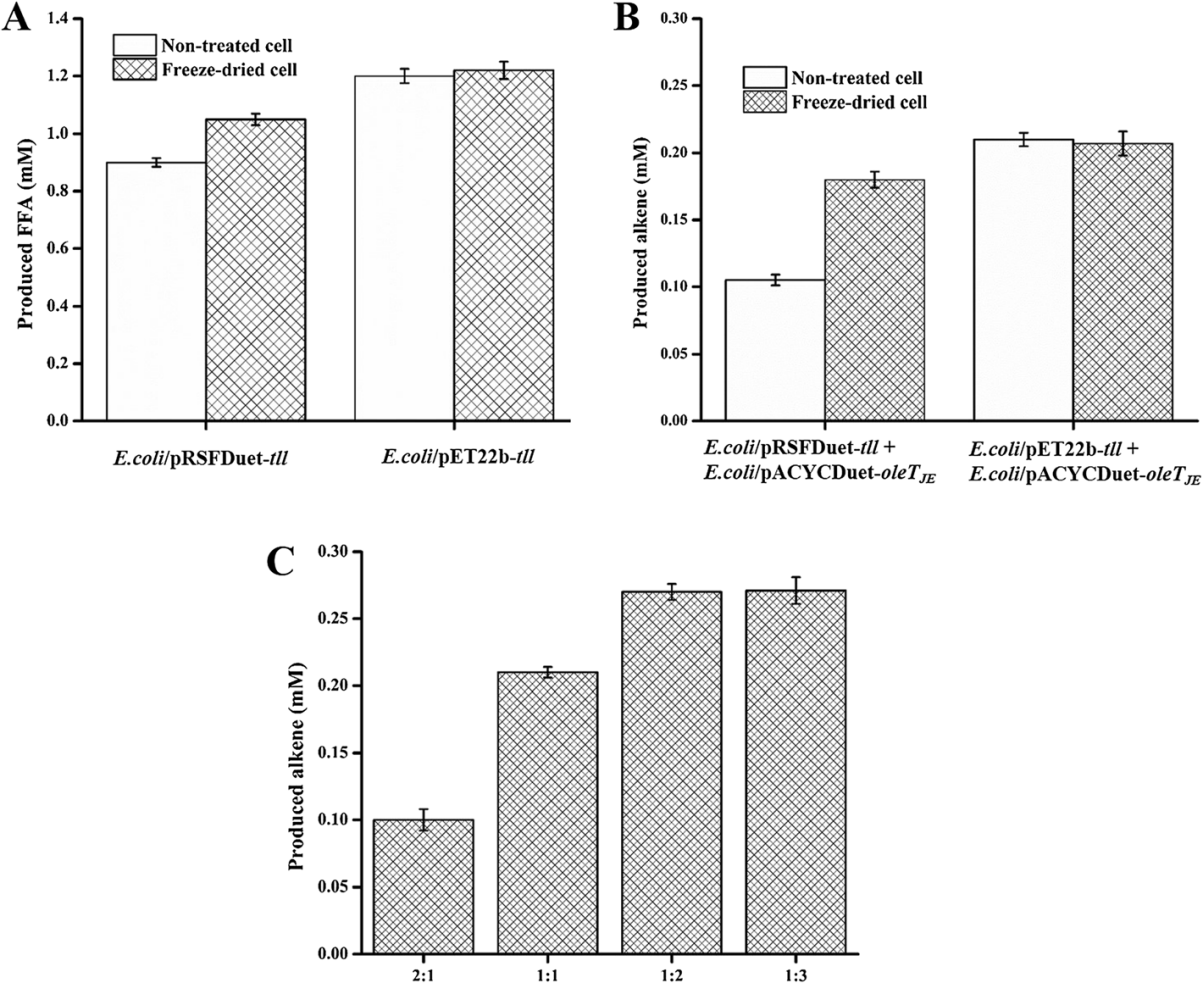
**Production of FFAs and 1-alkenes from microalgae oil and optimization of enzyme ratio. (A)** Production of FFAs from microalgae oil by resting *E. coli* cells with cytoplasmic and periplasmic overexpression of Tll. **(B)** Production of 1-alkenes from microalgae oil catalyzed by mixed resting *E. coli* cells. **(C)** Optimization of the enzyme ratio between Tll and OleT_JE_ in the form of freeze-dried whole cell catalysts for improvement of the overall alkene yield from microalgae oil.

For the TAG hydrolytic reactions mediated by overexpressed cytoplasmic Tll in *E. coli*/pRSFDuet-*tll* whole cells, freeze-dried cells hydrolyzed 70% microalgae oil to form 1.05 mM FFAs (Figure 3A), which are more catalytically efficient than non-treated cells (0.9 mM, 60 ± 2.3%). This indicates freeze-drying is a simple but effective way to improve substrate accessibility and hence catalytic activity.

To further improve mass transfer of TAG substrates, Tll was periplasmically overexpressed in *E. coli*/pET-22(b)-*tll* in order to hydrolyze TAGs more efficiently. As expected, a higher yield of 80% (1.2 mM FFAs produced) was achieved (Figure 3A). For periplasmic overexpression of Tll in *E. coli*, fusion of the Tll lipase to the pelB leader sequence directs the lipase to *E. coli* periplasm, where the leader peptide is removed by a signal peptidase [24]. Considering that the two types of cells used for biotransformations contained the same total activities (normalized in terms of cell-free extract), we reason that the enhancement of FFA production catalyzed by periplasmic Tll overexpression cells was likely attributed to improved substrate accessibility by localization of enzymes in periplasm. The freeze-dried *E. coli*/pET-22(b)-*tll* barely increased the degree of hydrolysis (Figure 3A).

In the case of Tll-OleT_JE_ coupled reaction in the mixed cell system, *E. coli*/pET-22(b)-*tll* (for periplasmic expression of Tll) plus *E. coli*/pACYCDuet-*oleT*_*JE*_ (for cytoplasmic expression of OleT_JE_) gave higher alkene yield (0.21 mM, 14 ± 0.4%) than that (0.10 mM, 7 ± 0.3%) of *E. coli*/pRSFDuet-*tll* (for cytoplasmic expression of Tll) plus *E. coli*/pACYCDuet-*oleT*_*JE*_ (for cytoplasmic expression of OleT_JE_) (Figure 3B). Also shown in Figure 3B, the mixed freeze-dried cells displayed higher conversion than the non-treated counterparts for the group with cytoplasmically overexpressed Tll (*E. coli*/pRSFDuet-*tll* + *E. coli*/pACYCDuet-*oleT*_*JE*_), but there was only a slight improvement for the group with periplasmic overexpression of Tll (*E. coli*/pET-22(b)-*tll* + *E. coli*/pACYCDuet-*oleT*_*JE*_).

Further optimization of the enzyme ratio in the form of freeze-dried cells (*E. coli*/pET-22(b)-*tll* + *E. coli*/pACYCDuet-*oleT*_*JE*_) enhanced the yield to 18 ± 0.8% (0.27 mM) at a 1:2 molar ratio (Tll:OleT_JE_) (Figure 3C). Although the ratio between the two enzymes can be adjusted in the mixed cell system, the catalytic efficiency of the whole system apparently would suffer from complicated transportation of intermediate FFAs from one type of cells to another. This is likely the major reason for the low conversions from TAGs to 1-alkenes. Therefore, engineering of an *E. coli* strain with co-expression of the two enzymes seems necessary.

### Biosynthesis of 1-alkenes with a whole cell catalyst co-expressing Tll and OleT_JE_

**Figure 4:**
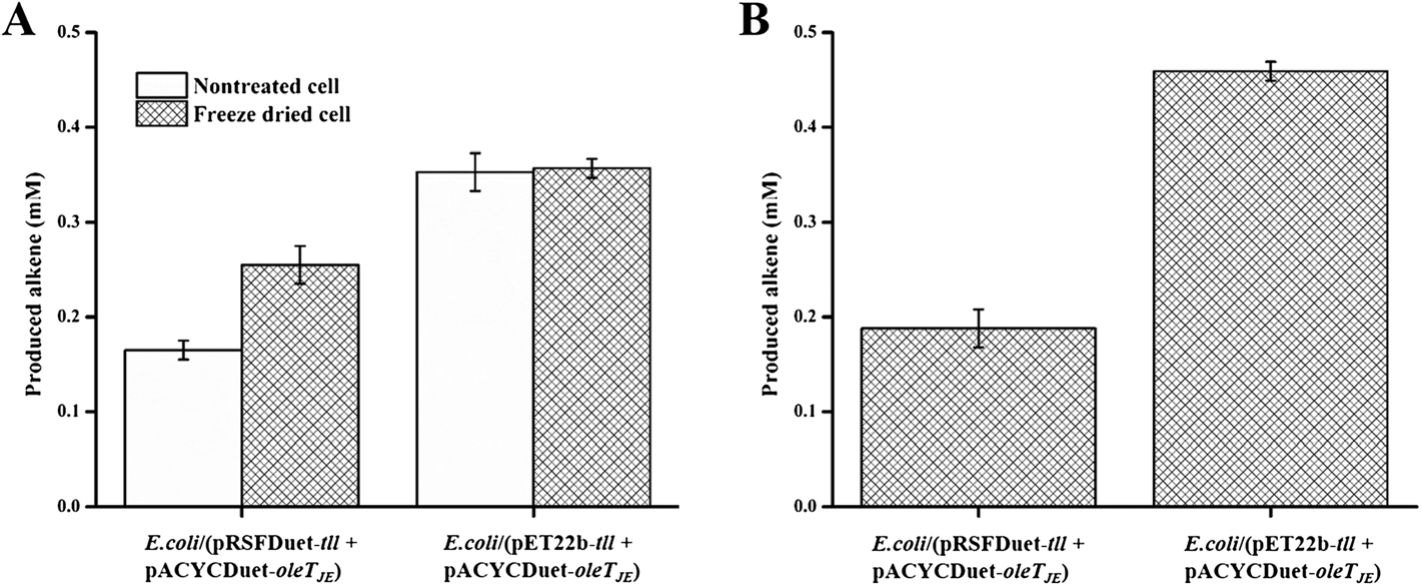
**Production of 1-alkenes from microalgae oil. (A)** Production of 1-alkenes from microalgae oil catalyzed by *E. coli* resting cells with co-expression of Tll and OleTJE. **(B)** Production of 1-alkenes from microalgae oil catalyzed by growing *in situ E. coli* cell catalysts.

The approach of co-expressing Tll (for oil hydrolysis) and OleT_JE_ (for alkene formation) in the same *E. coli* host could potentially overcome complex cross-membrane transport of intermediate FFAs between different types of cells, thereby gaining more efficient substrate channeling. Thus, we constructed two types of co-expression strains including *E. coli*/pRSFDuet-*tll* + pACYCDuet-*oleT*_*JE*_ and *E. coli*/pET-22(b)-*tll* + pACYCDuet-*oleT*_*JE*_ and evaluated their alkene-producing abilities. The non-treated *E. coli* cells with cytoplasmic co-expression of Tll and OleT_JE_ (*E. coli*/pRSFDuet-*tll* + pACYCDuet-*oleT*_*JE*_) converted 11 ± 0.7% microalgae oil into 1-alkenes (0.16 mM), whereas the freeze-dried cells gave a higher yield of 17 ± 1.1% (0.26 mM) (Figure 4A), both of which are greater than that of corresponding mixed cell systems. This improvement can be explained by better channeling of intermediates between adjacent enzyme active sites because of co-localization of two enzymes inside a single cell.

A similar profile was observed for the periplasmic co-overexpression cell system (*E. coli*/pET-22(b)-*tll* + pACYCDuet-*oleT*_*JE*_). Specifically, the strain of *E. coli*/pET-22(b)-*tll* + pACYCDuet-*oleT*_*JE*_ demonstrated improved hydrocarbon yield (0.35 mM, 24 ± 1.3%) compared to the corresponding cytoplasmic overexpression cell system (*E. coli*/pRSFDuet-*tll* + pACYCDuet-*oleT*_*JE*_). Again, this result indicates the importance of the contact between TAGs and Tll for the whole alkene-producing pathway. The freeze-dried periplasmic overexpression cells (*E. coli*/pET-22(b)-*tll* + pACYCDuet-*oleT*_*JE*_) did not enhance the conversion (0.36 mM, 24 ± 1.4%) (Figure 4A), indicating that freeze-drying treatment cannot further improve the cell permeabilization that has already been optimized by periplasmic expression of Tll. We infer that periplasmic expression of Tll probably leads to better permeability than the corresponding cytoplasmic expression; thus, the freeze-drying treatment showed a positive permeabilization effect on the cytoplasmic co-expression cell type, but not on the periplasmic co-expression type.

Measurement of the activity of the two enzymes contained in co-expression cells showed molar ratios of 1:1.7 and 1:1.9 (Tll:OleT_JE_), respectively, close to the optimal ratio of 1:2. More significantly, the periplasmic co-expression cells retained more than 70 ± 3.2% alkene-producing activity after recycling in three successive batches, displaying great potential for industrial application.

### Biosynthesis of 1-alkenes by co-expressed Tll and OleT_JE_*in situ* during cell growth

Integration of enzyme generation and enzymatic transformation into a single process would be significantly more energy efficient and of industrial simplification by making better use of fermentation energy and skipping enzyme purification. We previously developed an integrated bioprocess for *in situ* biodiesel production that occurs simultaneously with lipase generation in a *Pichia pastoris* yeast system [25]. Inspired by this strategy, in the present study, in parallel with combining hydrolysis of TAGs and decarboxylation of FFAs into a single *E. coli* host, we tested the strategy of developing *E. coli in situ* catalytic system for biosynthesis of 1-alkenes from TAGs. This *in situ* system integrated the process of enzyme production with the enzyme-catalyzed tandem biotransformations into a single process in one pot, which could significantly save energy consumption and simplify operational procedures.

Based on the tandem hydrolysis-decarboxylation *in situ* catalyzed by Tll and OleT_JE_ during cell growth, the two types of Tll-OleT_JE_ co-expression *E. coli* cells (*E. coli*/pRSFDuet-*tll* + pACYCDuet-*oleT*_*JE*_ and *E. coli*/pET-22(b)-*tll* + pACYCDuet-*oleT*_*JE*_) gave 0.19 mM (12 ± 0.6%) and 0.46 mM (31 ± 1.1%) alkene yield from the fed microalgae oils, respectively (Figure 4B). Interestingly, periplasmic overexpression cells (*E. coli*/pET-22(b)-*tll* + pACYCDuet-*oleT*_*JE*_) even secreted some lipases into the extracellular space (that is, culture medium) during the *in situ* process, which may significantly facilitate the hydrolysis-decarboxylation coupled reactions, as reflected by enhanced alkene production. Thus, the *in situ* catalytic system based on periplasmic overexpression could take advantage of both cell-free enzymes and whole cell catalysts. The extracellular and intracellular enzymes of growing *E. coli* cells were likely to be simultaneously utilized to produce alkenes *in situ* and in one pot. Moreover, we further evaluated this *in situ* system by recovering the whole cells and applied them for a new batch reaction as resting whole cell catalysts, which displayed 61.0 ± 2.2 and 73.0 ± 2.9% relative yield for cytoplasmic overexpression cells (*E. coli*/pRSFDuet-*tll* + pACYCDuet-*oleT*_*JE*_) and periplasmic overexpression cells (*E. coli*/pET-22(b)-*tll* + pACYCDuet-*oleT*_*JE*_), respectively.

Notably, higher concentrations of the substrate oil (1 to 5 mM) or H_2_O_2_ (1 to 15 mM) were also tested; however, the alkene yields did not increase (data not shown). It is possible that the low activity or low tolerance of OleT_JE_ toward the oil-FFA mixture may limit higher alkene yield. Thus, based on the crystal structure of OleT_JE_ recently reported by Belcher et al. [26], the protein engineering of this enzyme to improve its activity or tolerance in the context of two-step reactions could be helpful for developing a more efficient converter microorganism.

**Figure 5:**
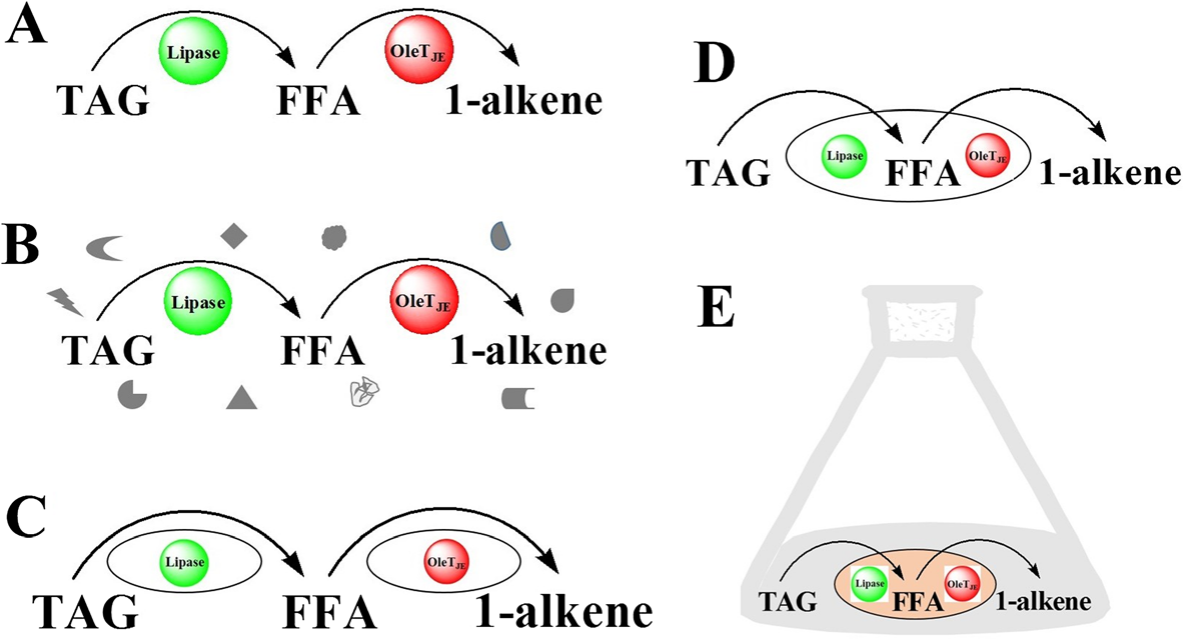
**Catalytic systems for the conversion of triacylglycerol to 1-alkene.** Various lipase-OleT_JE_ coupling catalytic systems for the conversion of triacylglycerol to 1-alkene including **(A)** purified enzymes, **(B)** cell-free extract, **(C)** mixed resting cells, **(D)** co-expression cells, and **(E)** *in situ* growing cells. FFA, free fatty acid; TAG, triacylglycerol.

Compared to the *in vivo* construction and regulation of metabolic pathways, the *in vitro* strategy of cell-free enzyme-based and whole cell-based systems represents an important opportunity for bioproduction of hydrocarbons. These *in vitro* systems could be readily controlled through preparation of enzyme or whole cell cocktails by simple mixing procedures or accurate modification of reaction conditions [27,28]. These multiple cell-free enzyme systems (purified enzymes, cell-free extracts) and cell-based systems (mixed resting cells, whole cells with co-expressed enzymes, growing *in-situ* cell catalysts) provide more flexible choices for specific purpose and situation (Figure 5).

## Conclusion

A novel biosynthetic pathway for production of biohydrocarbon 1-alkenes from renewable oils was assembled, which consists of the lipase Tll to release FFAs from TAGs and the P450 fatty acid decarboxylase OleT_JE_ responsible for decarboxylation of FFAs to generate 1-alkenes. The biotransformation of TAGs **→** FFAs **→** 1-alkenes was achieved using a variety of biocatalytic systems, including purified enzymes, cell-free extracts, resting whole cells, and growing *in situ* cell catalysts. These one-pot tandem biocatalyzes only required two sequential enzymatic steps, but allowed the efficient production of 1-alkenes from TAGs with 6.7 to 46.0% conversion ratios from 0.5 mM renewable feedstock including pure TAGs and diverse natural oils. Especially, the biosynthesis of 1-alkenes based on resting whole cells and growing *in situ* cell catalysts exhibits potential for industrial application.

Finally, it is worth noting that this novel artificial biosynthetic route could be adapted for other microorganisms, especially for those genetically tractable oleaginous microorganisms if considering the abundant endogenous TAGs as potential cellular substrates for the Tll-OleT_JE_ coupled system.

## Materials and methods

### Plasmids, strains, and reagents

The plasmids pACYCDuet-1, pRSFDuet-1, and pET-22(b) were obtained from Novagen (Darmstadt, Germany). Recombinant plasmid pET-28(b)-*oleT*_*JE*_ for expression of the P450 fatty acid decarboxylase OleT_JE_ was constructed in our laboratory previously [10]. *E. coli* DH5α and BL21 (DE3) strains were preserved in our laboratory. Various authentic standards of TAGs (C12, trilaurin, C14, trimyristin, and C16, tripalmitin), fatty acids (lauric acid, myristic acid, palmitic acid, stearic acid, and heptadecanoic acid), and fatty 1-alkenes (1-undecene, 1-tridecene, 1-pentadecene, and 1-heptadecene) were purchased from TCI (Shanghai, China). Kanamycin, chloramphenicol, thiamine, and isopropyl β-d-1-thiogalactopyranoside (IPTG) were products of Solarbio Science & Technology Co., Ltd (Beijing, China). All used restricted enzymes were supplied by Thermo Scientific (Shanghai, China). PrimeSTAR GXL DNA polymerase and dNTPs were from Takara Bio Inc. (Otsu, Japan). DNA manipulation kits were bought from Omega Bio-Tek (Norcross, GA, USA) and Promega (Madison, WI, USA). Ni-NTA resin was from Qiagen (Venlo, Netherlands). PD-10 desalting columns were products of GE Healthcare (Pewaukee, WI, USA). Ultra centrifugal filters were purchased from Millipore (Billerica, MA, USA). Bradford Protein Assay kit was bought from Beyotime Institute of Biotechnology (Jiangsu, China). Other routine reagents were commercially available products of analytical grade. The *tll* gene was synthesized by GenScript (Piscataway, NJ, USA). Oligo primers were synthesized by Sangon Biotech (Shanghai, China). Soybean oil and olive oil were purchased from local market. Microalgae oil was kindly donated by Professor Tianzhong Liu at the Qingdao Institute of Bioenergy and Bioprocess Technology, Chinese Academy of Sciences.

TB medium was composed of 1.2% tryptone, 2.4% yeast extract, 0.5% glycerol, 0.23% KH_2_PO_4_, and 1.25% K_2_HPO_4_. One millimolar of thiamine was supplemented for the expression of OleT_JE_. For maintaining corresponding plasmids, 25 μg/ml chloramphenicol or 50 μg/ml kanamycin was added.

The lysis buffer (pH 8.0) was composed of NaH_2_PO_4_ 50 mM, NaCl 300 mM, glycerol 10%, amd imidazole 10 mM. The washing buffer (pH 8.0) comprised NaH_2_PO_4_ 50 mM, NaCl 300 mM, glycerol 10%, and imidazole 20 mM. Elution buffer (pH 8.0) was composed of NaH_2_PO_4_ 50 mM, NaCl 300 mM, glycerol 10%, and imidazole 250 mM. Desalting buffer (pH 7.4) was a mixture of NaH_2_PO_4_, EDTA 1 mM, and glycerol 10%.

### Cloning and expression of enzymes

The *oleT*_*JE*_ gene was amplified using the previously constructed plasmid pET-28(b)-*oleT*_*JE*_ as template and the primer pair as follows: *Bam*HI-*oleT*_*JE*_, CGC*GGATCC*GATGGCAACACTTAAGAGGGATAAGGGCTTA (the *Bam*HI restriction site is italicized); and *Hin*dIII-*oleT*_*JE*_, CAATG*AAGCTT*TTATGTTCTGTCTAC AACTTCGC (the italicized nucleotides denote the *Hind*III cutting site)*.* For *tll* gene cloning, the synthetic *tll* gene (Genbank accession number AF054513.1) was used as template for PCR amplification with primers including *Bam*HI-*tll*, AGCCA*GGATCC*GAGTCCTATTCGTCGAGAGGTCTCG and *Hind*III-*tll*, GCCGC*AA GCTT*CTAAAGACATGTCCCAATTAACCC. These amplified *oleT*_*JE*_ and *tll* fragments were double digested with *Bam*HI/*Hind*III and ligated into pACYCDuet-1 and pRSFDuet-1 to create the recombinant plasmids pACYCDuet-*oleT*_*JE*_ and pRSFDuet-*tll*, respectively. The recombinant plasmids were first transformed into competent cells of *E. coli* DH5α for DNA sequence confirmation, and then transformed into *E. coli* BL21 (DE3) for cytoplasmic overexpression of OleT_JE_ and Tll. For construction of engineered *E. coli* strains with periplasmic overexpression of lipase, the plasmid pET-22(b) carrying an N-terminal *pelB* signal sequence was used. The *tll* fragment from *Bam*HI/*Hind*III double digested pRSFDuet-*tll* was inserted into corresponding sites of pET-22(b), resulting in the recombinant plasmid pET*-*22(b)-*tll*. The engineered *E. coli* BL21 (DE3) strains harboring corresponding recombinant plasmids were similarly constructed as described above.

A single colony of the *E. coli* BL21 (DE3) strain carrying pACYCDuet-*oleT*_*JE*_, pRSFDuet-*tll*, or pET-22(b) based counterparts were used to inoculate the seed culture in LB, and this culture was grown overnight at 37°C, 200 rpm. A 1% volume of pre-culture was inoculated into 1 l of TB medium with appropriate antibiotics (plus 0.5 mM 5-aminolevulinic acid and 1 mM thiamine for OleT_JE_ expression). When OD_600_ reached approximately 0.8, IPTG was added to the final concentration of 0.2 mM for induction of enzyme expression at 18°C for 20 h.

### Purification of enzymes

The harvested post-induced cells by centrifugation were resuspended with 40 ml lysis buffer and disrupted using ultra-sonication. Cell slurry was separated into cell-free lysate and cell debris at 10,000 × *g* for 30 min at 4°C. The resulted cell-free lysate was gently mixed with 1 ml 50% Ni-NTA slurry at 4°C for 1 h. The mixture was loaded onto a column and washed with 100 to 200 ml washing buffer until no protein eluted in the flow-through. The 6 × His tagged proteins bound to Ni-NTA resin were eluted with 5 ml elution buffer. The eluted protein fraction was concentrated using Millipore ultrafilter with 30-kDa molecular weight cutoff (2,000 × *g*, 30 min, 4°C) and loaded onto a pre-equilibrated PD-10 column for buffer exchange using desalting buffer. All protein fractions were monitored using Bradford Protein Assay kit. The aliquots of collected fractions were flash frozen by liquid nitrogen and stored at −80°C for later use.

### Determination of enzyme concentration

Diluted purified OleT_JE_ solution was reduced by adding sodium dithionite prior to the initial scan at 400 to 600 nm to record the baseline spectrum. Then, the P450 enzyme solution was subjected to CO bubbling for 40 s before the second scan to get the reduced CO-bound P450 spectrum, based on which the functional OleT_JE_ was quantified from the absorbance difference between *A*_450_ and *A*_490_ using the molar extinction coefficient of 91 mM^−1^ cm^−1^ [29]. The lipase activity was measured as described elsewhere [25].

### Whole cell catalyst preparation

The induced culture broth was centrifuged to harvest fresh wet cells, termed as non-treated cells. These fresh wet cells were subjected to freezing at −80°C for 12 h, and then freeze-drying for 24 h with lyophilizer to obtain freeze-dried cells.

### Enzymatic assays and whole cell biotransformation

For cell-free enzyme biotransformation, the standard 100-μl hydrolysis reaction assay containing 0.5 mM substrate_,_ 2 μM purified Tll in sodium phosphate buffer (100 mM, pH 7.4) was carried out in a 1.5-ml Eppendorf tube at 30°C for 6 h. The tested substrates included pure TAGs (fatty acyl chain length of C12, C14, and C16) and oils (microalgae oil, soybean oil, and olive oil). For the tandem reactions of hydrolysis followed by decarboxylation, 2 μM OleT_JE_ and 1 mM H_2_O_2_ were supplemented into the hydrolysis reaction while maintaining the total volume of 100 μl. During optimization of the molar ratio between these two enzymes, the amount of each enzyme was adjusted appropriately.

For resting whole cell biotransformation, various amounts of resting recombinant cells (approximate 50 mg) with the same activities (in terms of cell-free extract) were added into 500 μl sodium phosphate buffer containing 0.5 mM microalgae oil. In the case of lipase-OleT_JE_ coupled catalysis reaction, 1 mM H_2_O_2_ was supplied. During optimization of the enzyme ratio in the whole cell catalysis, the cell amounts were accordingly adjusted. In the whole cell-mediated recycling biotransformations, *E. coli* cells were recovered and washed after each batch reaction, and then applied in a new batch reaction. These resting whole cell-mediated biotransformations were performed at 30°C for 24 h.

For biotransformation mediated by growing cells *in situ*, 12-h post-induction cultures were supplemented with 0.5 mM microalgae oil and 1 mM H_2_O_2_ for additional 36-h reaction in shaking flask at 28°C. All experiments were repeated three times.

### Determination of FFA and 1-alkene

The reactions were quenched and extracted with 100 μl ethyl acetate containing 0.5 mM heptadecenoic acid as internal standard for GC-MS analysis. The GC oven equipped with the HP-INNOWAX (Agilent Technologies, Inc., Santa Clara, CA, USA; 30 m × 0.25 mm × 0.25 μm) column was heated using the program as follows: isothermal at 40°C for 4 min, 40 to 250°C at the rate of 10°C/min, and 250°C for 15 min. The mass fragment range of 50 to 500 m/z was monitored under electron ionization conditions (1741 eV). FFAs and alkenes were quantified using corresponding standard compounds and the internal standard as references.

## Additional files

Additional file 1:
**Graphical abstract. An artificial two-step biosynthetic pathway for biological production of 1-alkenes using three kinds of natural oils.**
Additional file 2: Figure S1. Plasmid construction and purification of Tll (A) and OleT_JE_ (B). Additional file 3: Table S1. Profiles of produced FFAs via lipase Tll mediated hydrolysis and produced 1-alkenes via lipase-OleT_JE_ tandem biotransformation. All experiments were performed three times.

## Abbreviations

TAGs: triacylglycerols
Tll: *Thermomyces lanuginosus* lipase
FFAs: free fatty acids
IPTG: isopropyl β-d-1-thiogalactopyranoside
