# Supplementary figures and images for: Assembly of lipase and P450 fatty acid decarboxylase to constitute a novel biosynthetic pathway for production of 1-alkenes from renewable triacylglycerols and oils
Source: Biotechnol Biofuels. 2015 Feb 26;8:34. doi: 10.1186/s13068-015-0219-x (PMC4355466; doi:10.1186/s13068-015-0219-x)

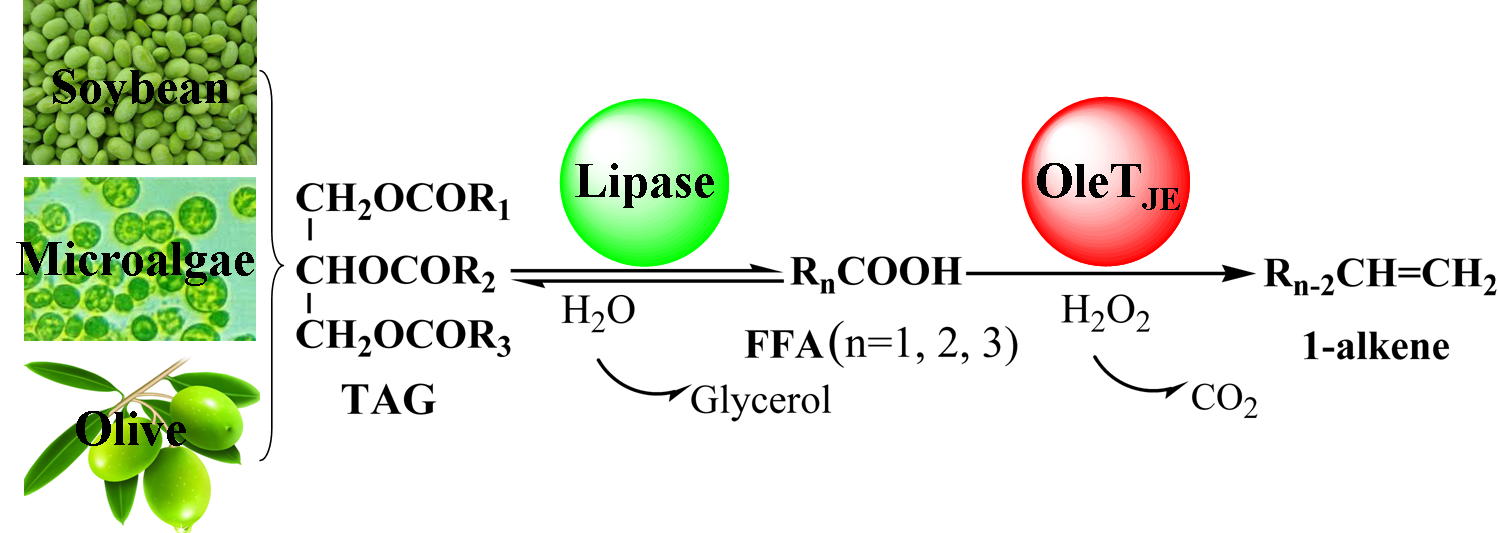

Supplement: Additional file 1: — Graphical abstract. An artificial two-step biosynthetic pathway for biological production of 1-alkenes using three kinds of natural oils. [file 13068_2015_219_MOESM1_ESM.tiff]

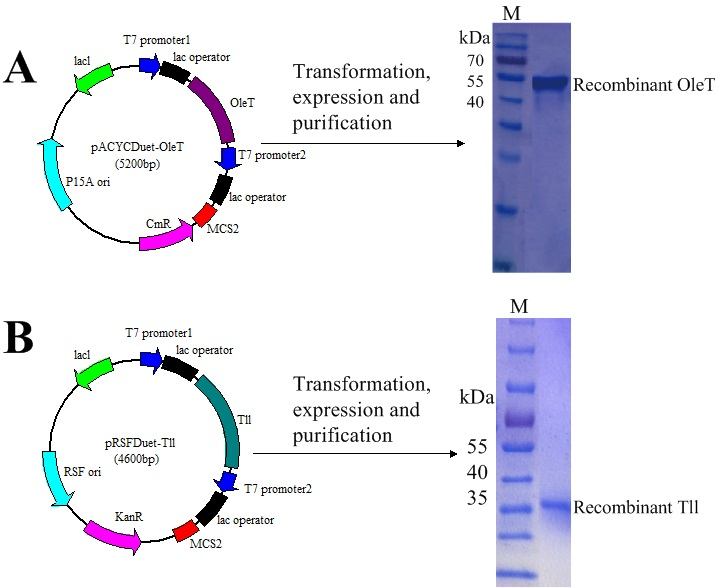

Supplement: Additional file 2: Figure S1. — Plasmid construction and purification of Tll (A) and OleTJE (B). [file 13068_2015_219_MOESM2_ESM.tiff]
