# Supplementary material for: Assembly of lipase and P450 fatty acid decarboxylase to constitute a novel biosynthetic pathway for production of 1-alkenes from renewable triacylglycerols and oils
Source: Biotechnol Biofuels. 2015 Feb 26;8:34. doi: 10.1186/s13068-015-0219-x (PMC4355466; doi:10.1186/s13068-015-0219-x)
Supplement: Additional file 3: Table S1. — Profiles of produced FFAs via lipase Tll mediated hydrolysis and produced 1-alkenes via lipase-OleTJE tandem biotransformation. All experiments were performed three times. [file 13068_2015_219_MOESM3_ESM.pdf]

Oil feedstock	Produced FFA composition (% of produced total FFA)								Produced total FFA (mM)	Produced alkene (mM)		
	C16:0	C16:1	C18:0	C18:1	C18:2	C18:3	C20:5	C22:6		C13	C15	C17
Olive oil	19.91 ±0.18	0.42 ± 0.03	13.12 ±0.15	58.01 ±0.16	6.49 ± 0.09	2.05 ± 0.07	-	-	1.21 ±0.09	-	0.21 ± 0.07	0.13 ± 0.05
Soybean oil	15.16 ±0.11	0.59 ± 0.03	16.98 ±0.14	45.52 ±0.24	20.82 ±0.13	0.93 ± 0.06	-	-	1.12 ±0.07	-	0.15 ± 0.03	0.15 ± 0.05
Microalgae oil	20.54 ±0.31	0.12 ± 0.02	20.56 ±0.40	19.78 ±0.13	15.25 ±0.12	15.36 ±0.15	2.53 ± 0.07	5.86 ± 0.08	1.37 ±0.04	0.08 ± 0.01	0.20 ± 0.02	0.20 ± 0.01
